# Savings from biosimilars and Medicare formulary access

**DOI:** 10.1093/haschl/qxaf214

**Published:** 2025-11-12

**Authors:** Kirsten Axelsen, Delphine Courmier, Ge Bai, Luca Maini

**Affiliations:** DLA Piper, Policy Advisor, New York, NY, 10020, United States; Organon, Global Pricing and Market Access, Jersey City, NJ, 07302 United States; Johns Hopkins University, Carey Business School, Washington DC, 21202 United States; Harvard University, Medical School, Boston, MA, 02118 United States

**Keywords:** biosimilars, formulary, Medicare Part D, drug costs

## Abstract

We reflect on how Medicare Part D formulary design could facilitate access to biosimilars, create cost-savings, and motivate investment in biosimilar development. We evaluated adalimumab, a self-administered biologic for inflammatory conditions with the most approved biosimilars, including interchangeable as a case study. We found that formulary access in Part D for adalimumab biosimilars is lower relative to the innovator. Moreover, when adalimumab biosimilars are on the formulary, beneficiaries typically pay coinsurance, a cost higher than a fixed copay. This is unlike generic drugs, whose coverage in most Part D formularies typically has a co-pay of $5 or less (Dusetzina SB, Cubanski J, Nshuti L, et al. Medicare Part D plans rarely cover brand-name drugs when generics are available. *Health Aff [Millwood]*. 2020;39[8]:1326-1333. https://doi.org/10.1377/hlthaff.2019.01694). The US biosimilar market has been slow to grow, limiting potential cost-savings. The investment required to develop and manufacture a biosimilar is larger than that for a generic and requires a greater return for investment, and many off-patent biologics lack a biosimilar competitor (Arad N, Staton E, Hamilton M, et al. *Realizing the Benefits of Biosimilars: Overcoming Rebate Walls*. Duke Margolis Center for Health Policy; 2022). The Food and Drug Administration's October 2025 effort to simplify evidence requirements for biosimilarity may reduce development costs, but without market access financial returns from development are limited (Food and Drug Administration. FDA moves to accelerate biosimilar development and lower drug costs [press release]. October 29, 2025. https://www.fda.gov/news-events/press-announcements/fda-moves-accelerate-biosimilar-development-and-lower-drug-costs). Policies to encourage formularies to expand access to biosimilars in programs such as Medicare could motivate investment.

## Introduction

In the face of continued growth, US policymakers seek approaches to reduce drug costs. Increasing biosimilar adoption through more favorable formulary access is an opportunity for near-term savings while encouraging investment in biosimilar development for long-term cost management. Spending on biologic drugs is growing more rapidly than chemical-based drugs; as of 2023, biologics account for 45% of US drug spending.^1^ This contributes to growing premiums in Medicare, and costs for beneficiaries and government.

Medicines with different molecular composition have distinct pathways to competitive entry, with biosimilar approval for biologic drugs made from living cells and generic drug approval for chemical-based medicines. A biosimilar is a medication that has no clinically meaningful differences and is similar to a Food and Drug Administration (FDA)–approved biologic. A generic drug can be created to be nearly the same as a chemical-based medication. Generic medications are often substituted for a branded drug directly by the pharmacy without consulting a health care provider. Biosimilars are not automatically substituted unless they receive an interchangeable designation, which requires submitting clinical trial evidence to the FDA.

Generic medicines replace 75% or more of the originator in 1 year, compared with 15%-40% for biosimilars.^2^ Unlike generics, where all are typically covered, health plans may choose to exclude all or certain biosimilars from their formulary. This may be done to seek rebates from the biologic drug manufacturer for formulary placement.^3^

Biologics losing patent protection in 2025-2034 account for $234 billion, but only 23% of current innovator biologics without patent protection have a biosimilar on the market, while 10% have a biosimilar in development, potentially due to lack of anticipated return on investment from an approved biosimilar.^1^ This lack of impeding competition limits savings generated when the biologic loses market exclusivity.

Drug plans set Medicare Part D formularies and can encourage use of self-administered medicines by placing drugs on formulary, where lower tiers cost the beneficiary less. Making biosimilars more affordable on those formularies would increase use and revenues that attract investment in biosimilar development. Moreover, it is the largest and costliest part of the Medicare drug benefit, with many biologics facing biosimilar competition.^4^ Medicare Part B, for physician-administered drugs, has nearly uniform 20% coinsurance so there is less ability to use cost-sharing to guide beneficiary selection.

To illustrate biosimilars’ formulary challenges, we examined adalimumab, a self-administered biologic drug with 10 approved biosimilars, the most of any biologic.^5^ We evaluated formulary access in Medicare Part D plans between December 2023 and July 2025 (Table 1). We considered cost and use in Medicare Part D in 2023, the most recent year of Centers for Medicare and Medicaid Services (CMS) data available (Table 2).

**Table 1.** Medicare Part D formulary access to adalimumab and approved biosimilars.

				% on formulary			% tier 3 or below		
Molecule	Brand	Biosimilar FDA approval date	Biosimilar FDA interchangeable designation date	December 2023 (a)	December 2024 (a)	July 2025 (a)	December 2023 (a)	December 2024 (a)	July 2025 (a)
Adalimumab	Humira	N/A	N/A	98.7	90.7	74.5	0.7	0.8	0.8
Adalimumab-atto	Amjevita	September 2016	August 2024	2.4	2.7	3.8	67.9	67.0	85.6
Adalimumab-adbm	Cyltezo	August 2017	October 2021	52.5	47.2	64.0	0.3	0.6	0.3
Adalimumab-adaz	Hyrimoz	October 2018	April 2024	31.2	18.1	19.7	0.2	0.1	0.2
Adalimumab-bwwd	Hadlima	July 2019	June 2024	5.5	15.5	11.7	0.4	1.1	1
Adalimumab-afzb	Abrilada	November 2019	October 2023	0	1.8	3.2	0	0	0
Adalimumab-fkjp	Hulio	July 2020	February 2025	3.3	0.6	0.4	0.6	62.5	60
Adalimumab-aqvh	Yusimry	December 2021	N/A	1.8	0.4	0.2	0	0	0
Adalimumab-aacf	Idacio	December 2022	N/A	0	24	19.4	0	1.2	1.7
Adalimumab-aaty	Yuflyma	May 2023	April 2025	0	37.4	53.8	0	0.7	0.3
Adalimumab-ryvk	Simlandi	February 2024	February 2024	N/A	9.8	10.4	0	0.5	0.6

Data from references 5 and 6.

(a) Weighted average across all forms of the biologic or biosimilar.

Abbreviations: FDA, Food and Drug Administration; N/A, not applicable.

**Table 2.** Medicare Part D utilization of adalimumab and approved biosimilars in 2023.

		Full-year 2023		
Molecule	Brand	Total spending per beneficiary, Medicare^a^	No. of Medicare beneficiaries	Percentage of Medicare beneficiaries
Adalimumab	Humira	$65 906	91 972	96
Adalimumab-atto	Amjevita	$12 245	3296	3
Adalimumab-adbm	Cyltezo	$15 817	107	0
Adalimumab-adaz	Hyrimoz	$20 629	16	0
Adalimumab-bwwd	Hadlima	$2530	93	0
Adalimumab-afzb	Abrilada			
Adalimumab-fkjp	Hulio	$4059	11	0
Adalimumab-aqvh	Yusimry	$4057	2	0
Adalimumab-aacf	Idacio			
Adalimumab-aaty	Yuflyma	$9462	13	0

Data from reference 4.

^a^Undiscounted full-year cost to Medicare Part D per beneficiary using drug.

In December of 2023, only 2 biosimilars received coverage on more than 30% of Part D formularies: Cyltezo, an interchangeable biosimilar approved before 2003, and Hyrimoz. Despite this, both had low utilization. Only a third biosimilar, Amjevita, was used by more than 1% of Medicare beneficiaries in 2023, more than the interchangeable Cyltezo. In 2025, still only 2 were covered by most Part D formularies. Branded adalimumab, is the drug with the largest share of formulary coverage in 2025 and also accounted for $6 billion in 2023 gross Part D spending, or 99% of the total cost for adalimumab and its biosimilars in that year.^4,6^

Patients will default to the originator if biosimilar cost-sharing is not favorable. We found that, when biosimilars were covered, it was almost always in tier 4 or higher, where beneficiaries typically pay coinsurance rather than copays.^7^ Only Amjevita was placed on tier 3 or below on most formularies that covered the drug in 2023; its co-pays ranged from $43 to $45 in 2023 to 2025, where 99% of plans require coinsurance for Humira. In 2023, Humira's cost per claim for commonly used forms was $8500-$9200 or $2550-$2760 out-of-pocket with 30% coinsurance, the average rate for Humira. This exceeds the yearly cost of a $45 co-pay, which is offered on 68%-86% of the plans covering Amjevita ($540).^4,6^

In 2025, 99% of Part D formularies covered at least 1 adalimumab biosimilar, nearly always on a higher tier with the originator. So, beneficiary costs with the Medicare $2000 out-of-pocket cap would be nearly identical for the biologic and biosimilar.^8^ Moreover, biosimilars are not interchangeable with each other; those switching plans in 2025, when many plans exited, may not find access to their biosimilar.

## Conclusion

In 2023 to 2025, adalimumab biosimilars have more limited formulary coverage in Medicare Part D relative to the biologic, and cost-sharing is primarily coinsurance, not more affordable co-pays. We found limited uptake in 2023 for the approved biosimilars (1 interchangeable) with the largest being Amjevita, commonly offered in a lower tier, indicating that affordability may be influencing use, even though other biosimilars had more formulary coverage.

There may be opportunities to increase uptake of biosimilars through Medicare formulary management. These could include policies considered for generics, such as the $2 generic drug coverage model considered by Centers for Medicare and Medicaid Innovation, or increasing federal subsidies from 20% to 40% for biosimilars in catastrophic coverage.

While adalimumab may not be reflective of all biosimilars, it demonstrates the challenge to achieve formulary coverage, an existential threat to biosimilar development. Without coverage, biosimilars struggle to earn revenue, which reduces incentives to invest in biosimilar development and weakens future competition for biologics. As policymakers aim to reduce drug costs they should consider approaches to encourage plans to offer lower cost-sharing and greater formulary access for biosimilars in Part D.

## Supplementary Material

qxaf214_Supplementary_Data
